# Small intestinal autotransplantation for spontaneous isolated superior mesenteric artery dissection: A case report

**DOI:** 10.1097/MD.0000000000017837

**Published:** 2019-11-22

**Authors:** Jiangpeng Wei, Yi Yang, Jianyong Zheng, Dongli Chen, Weizhong Wang, Qingchuan Zhao, Xiaohua Li, Guosheng Wu

**Affiliations:** a Xijing Hospital of \Digestive Diseases; b Department of Radiology, Xijing Hospital, The Air Force Military Medical University, Xi’an, Shaanxi, People's Republic of China.

**Keywords:** autotransplantation, small intestinal arterial dissection

## Abstract

**Introduction:**

Spontaneous isolated superior mesenteric artery dissection (SISMAD) is a rare differential diagnosis for patients presenting with abdominal pain. Due to limited cases reported, surgical management strategies are poorly defined.

**Patient concerns:**

A 54-year-old man presented to our emergency department with a 4-day history of epigastric pain combined with nausea and vomiting. The pain was dull, constant, and unbearable. It was accompanied by abdominal distention, but there was no radiating pain, chills, fever, or hematochezia. The patient did not have a history of abdominal surgeries, or tobacco or illicit drug use.

**Diagnosis:**

A contrast-enhanced computerized tomography (CT) scan demonstrated an isolated and spontaneous superior mesenteric artery dissection with aneurysmal evolution of the false lumen, involving multiple side branches. The middle-lower jejunum and the whole ileum were extensively dilated, and the middle jejunum was ischemic with edema.

**Interventions:**

Exploratory laparotomy and autologous small bowel transplantation.

**Outcomes:**

The patient was successfully treated using exploratory laparotomy and intestinal autotransplantation (IATx) without bowel resection and had a stable recovery without complications.

**Conclusion:**

For patients with severe mesenteric ischemia or those who fail to respond to initial conservative treatment, IATx may be a reasonable treatment strategy.

## 1 Introduction

Spontaneous isolated superior mesenteric artery dissection (SISMAD) is defined as a dissection of the superior mesenteric artery (SMA) without concomitant alteration of the aorta, celiac artery, inferior mesenteric artery, or renal artery. It is a rare but potentially fatal arterial disease, and its incidence has been underestimated.^[1]^ Since the more widespread use of computed tomography (CT) scans, several cases were reported. However, there are no comprehensive reports on surgical indications and treatment modalities for SISMAD onset.^[2,3]^ Three possible therapeutic strategies for patients with SISMAD include conservative management with or without antithrombotic therapy,^[4–6]^ open surgery such as a bypass or direct surgical reconstruction of the SMA lesion,^[7,8]^ or endovascular therapy with SMA stenting.^[9–11]^ However, to date, intestinal autotransplantation (IATx) therapy for SISMAD has not been reported. Here, we present a case of SISMAD that was successfully treated with IATx.

## 2 Case report

**Figure 1:**
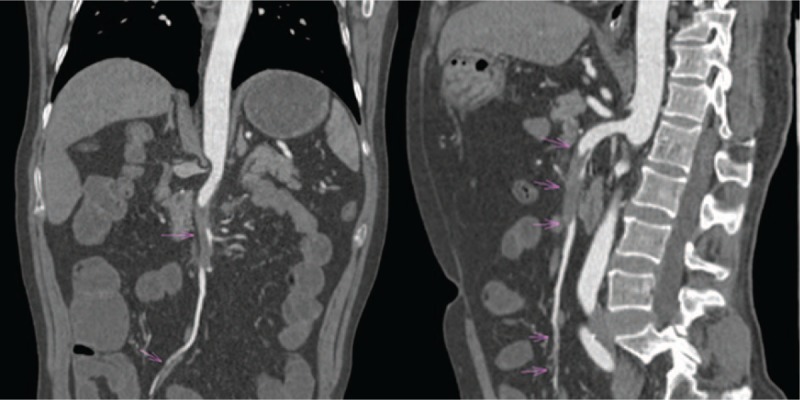
Computed tomography scan of the abdomen showing the area of superior mesenteric artery and ileocolic artery dissection with false lumen thrombosis (arrow).

**Figure 2:**
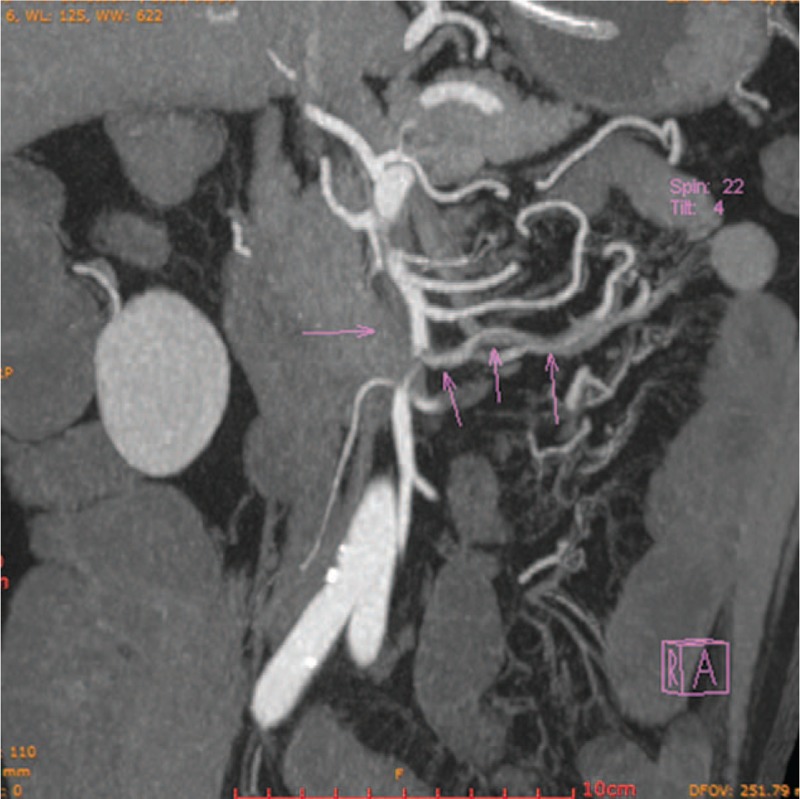
Computed tomography scan of the abdomen showing the left 2 branches of the superior mesenteric artery were appeared “low density filling defect sign,” and the true lumen were extremely constrictive (arrow).

**Figure 3:**
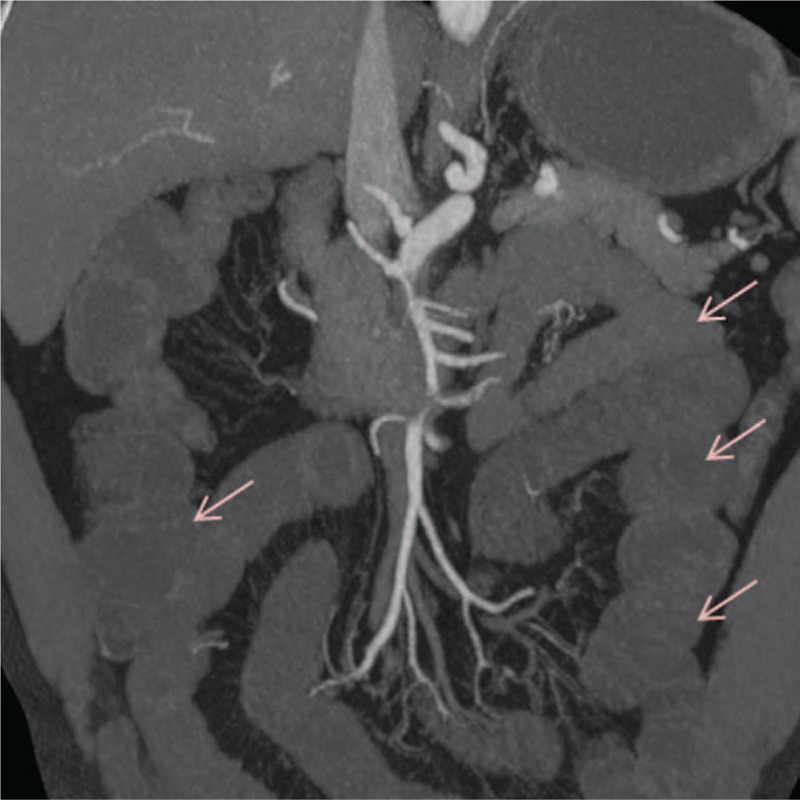
Coronary views of the small intestinal on enhanced computed tomography. Intestinal emphysema (arrow) was observed, suggesting intestinal necrosis.

A 54-year-old man presented to our emergency department with a 4-day history of epigastric pain combined with nausea and vomiting. The pain he experienced was dull, constant, and unbearable. It was accompanied by abdominal distention, but there was no radiating pain, chills, fever, or hematochezia. The patient did not have a history of prior abdominal surgeries, or tobacco, or illicit drug use. On arrival, blood pressure was 103/70 mmHg, heart rate was 90 beats per minute, and the body temperature was 36.7 °C. Physical examination revealed a whole abdominal tenderness, no signs of distended abdomen, but there were hypoactive bowel sounds. His white blood cell count was 19.17 × 10^9^, with 92% neutrophils. Other vital signs were in the normal range. A contrast-enhanced CT scan demonstrated an isolated and spontaneous SMA dissection with aneurysmal evolution of the false lumen, involving multiple side branches. The branches included an area of dissection with false lumen thrombosis in the proximal SMA beginning just beyond its origin for a length of about 6.5 cm (Fig. 1), the jejunal branch (the left 2 branches) of SMA showed signs of “low-density filling defect” (Fig. 2), and the true lumen was extremely constricted. Limited dissection could also be seen in the right inferior ileocolic artery (Fig. 1). The middle-lower jejunum and the whole ileum were extensively dilated, and the middle jejunum was ischemic with edema (Fig. 3). The multidisciplinary treatment team considered that the proximal segment of the SMA was dissected with apparent and long stenosis, as well as the full left 2 branches of SMA, while the right lower ileocolic artery had a localized dissection. Furthermore, the small intestine had become ischemic and endovascular therapy with SMA stenting or conservative management with antithrombotic therapy were considered complicated and of high risk. Therefore, we performed exploratory laparotomy and autologous small bowel transplantation. During the surgery, we found the majority of the small intestine to present with a reddish-brown color and chronic ischemic manifestations. The SMA was distended with cystic dilatation for about 6 to 7 cm from the lower edge of the pancreas to the distal end. The left 2 branches (jejunal branch) presented with distention cyst and the right inferior ileocecal artery showed localized cystic dilatation. According to the length of arterial dissection, an almost 2.7-m-long pre-transplanted small intestine (proximal resection line is about 1 m from Treitz ligament, distal resection line is about 0.4 m from ileocecal valve) was harvested. The pre-transplanted small intestine was successfully lavaged with organ preservation solution (1000 mL) at 4 °C. After the artery was disconnected from the graft, the time of warm ischemia was about 90 seconds. We performed an end-to-side anastomosis between the residual artery of the transplanted intestine and the aorta ventralis, and end-to-side anastomosis of the superior mesenteric vein and the inferior vena cava. The cold ischemic time was about 4 hours counted from the perfusion of the transplanted intestine to reperfusion after completion of the vascular anastomosis. Finally, the proximal and distal part of the transplanted intestine and the proximal and distal part of the residual small intestine were anastomosed side-by-side with staplers to complete the digestive tract reconstruction. Intraoperative ultrasound examination was used to detect the transplanted intestine and evaluate for thrombus near the vascular anastomosis. This was repeated 1 day after surgery using ultrasonography, which showed no signs of a thrombus. After the transplantation, the patient was moved to the intensive care unit for 1 day. He received low molecular weight heparin sodium therapy for the prevention of thrombosis. After 3 days of total parenteral nutrition, the patient developed ventilation and defecation, upon which enteral nutrition was started, which was gradually increased to a normal diet. On day 7 after surgery parenteral nutrition was discontinued, and the abdominal drainage tube was removed the next day. The patient had a stable recovery without complications and was discharged 13 days after surgery. For 3 years he was in follow up and remained in a good general condition.

## 3 Discussion

SISMAD is a rare differential diagnosis for patients presenting with abdominal pain.^[1]^ In an autopsy series, the incidence of SISMAD was reported to be 0.06%,^[12]^ this may, however, be an underestimation. Luan et al,^[13]^ reviewed the SISMAD literature published since 1947 and found it being reported more frequently in recent years. Presumed risk factors include atherosclerotic disease, hypertension, fibromuscular dysplasia, cystic medial necrosis, trauma, pregnancy, and connective tissue disorders.^[14,15]^ Physical examination findings include tenderness to palpation over the epigastric or left upper quadrant regions, and rarely, an audible epigastric bruit.^[16]^

Surgical interventions include artery ligation, endoaneurysmorrhaphy, aortomesenteric bypass, and laparotomic resection.^[2,4,5,11]^ A recent analysis including 514 cases showed that conservative treatment was safe and effective in >80% of symptomatic SISMAD cases, without apparent benefit of antithrombotic agent use. Initial or secondary intervention was more often endovascular, with favorable success rates in short-term outcomes.^[17]^ However, the optimal treatment strategy for this diagnosis remains controversial as the exact etiology and pathophysiological mechanisms of the disease have not been firmly established. Indications for surgical repair have previously been described as increasing aneurysmal dilation, thrombosis of the SMA true lumen, persistent symptoms despite anticoagulation,^[18]^ arterial rupture,^[19]^ or bowel infarction. Arterial rupture and bowel infarction are absolute indications for emergency surgical intervention, whereas the others are relative indications for the rare failure of medical management in symptomatic patients.^[20]^ No previous publications examined the incidence of SISMAD in patients with acute abdominal pain based on contrast-enhanced CT scans.^[21]^ In SISMAD patients, mesenteric anastomosis is technically demanding due to a thin dissected SMA wall, risk of distal or proximal progression of the arterial dissection, and difficult proximal control of the SMA.^[2]^ Endovascular treatment with SMA stenting has also been reported to result in a successful outcome but has a risk of complications such as arterial rupture or progression of arterial dissection, late development of in-stent restenosis, or thrombotic occlusion of the SMA stent.^[1]^

Therefore, despite the expected prompt efficacy of SMA stenting and conservative treatment, we recommend IATx for some patients with SISMAD, in particular when there are signs of acute bowel ischemia. Recently, partial pelvic exenteration, ex vivo resection, and IATx have been rendered to solve the obstacles to resection of symptomatic desmoid tumors.^[22–25]^ The physiologic basis of this technique is using a cold solution (University of Wisconsin solution) to create a prolonged time of intestinal cold ischemia status, averting irreversible damage, and allowing surgeons to remove the tumor with subsequent intestinal autotransplantation.^[25,26]^ This technique has several advantages, such as excellent exposure of the surgical field, optimum control of the critical step of tumor resection in a bloodless field, clear visualization of vascular pedicles, and arches to protect healthy tissues or ensure precise excision. According to the patency of the entry and re-entry sites, a simplified angiographic classification of SISMAD was established by Yu et al,^[27]^ which is based on Sakamoto classification.^[28]^ In this case, the patient's small intestine suffered from ischemic changes, and stent intervention, revascularization techniques, and IATx were all discussed by the multidisciplinary treatment team. First, endovascular therapy is suitable for symptomatic SISMAD patients with type II lesions. However, the current case was a type III, and it is challenging to find the site at which tearing of the artery wall started during dissection of the SMA when endovascular stent placement is performed. Second, revascularization techniques usually require either a thromboendarterectomy with direct arterial suture or using a patch, or a prosthetic aortomesenteric or venous bypass graft.^[29]^ The range of the blood vessel lesion is approximately 5 to 6 cm, which starts from the lower edge of the pancreas, and therefore, direct surgical reconstruction of the SMA lesion or thromboendarterectomy were anatomically and technically difficult for our center. Third, we did not consider the use of a prosthetic graft for aortomesenteric bypass because of concerns for long-term patency. Once failed, the patient will face the risk of resecting most of the small intestine, leading to short bowel syndrome.^[20]^

Therefore, we decided that IATx was the best strategy for this patient. Although IATx is associated with considerable operative risk, this aggressive approach allows patients with certain abdominal neoplasms involving the major mesenteric vessels to be resected entirely and attain early intestinal autonomy from parenteral nutrition.^[30]^ Combined with our experience in small bowel transplantation, we suggest that IATx surgery should be indicated for large size of a fusiform or saccular aneurysm, thrombosis of the true lumen of the dissection site, persistent symptoms despite anticoagulation, occlusive lesions jeopardizing the lower digestive tract, arterial complications such as rupture. In contrast, contraindications include effective conservative treatment, effective interventional therapy, no surgical experience of the treatment team in small bowel transplantation. However, this procedure remains technically challenging as it includes the involvement of multiple organs, prolonged operative time, and more blood transfusions. Thus, only a few case reports have described patients with mesenteric tumor encasing the blood supply of the small intestine, treated with ex vivo resection, and subsequent IATx.^[25,31,32]^

## 4 Conclusion

IATx can be therapeutic in SISMAD cases with acute or subacute small intestinal ischemia. For patients with severe mesenteric ischemia or those who fail to respond to initial conservative or endovascular treatment, IATx may be a reasonable option.

## Acknowledgments

The authors would like to thank the surgical teamand the nursing staff at the Xijing Hospital of Digestive Diseases, the The Air Force Military Medical University, for their excellent patient care.

## Author contributions

**Data curation:** Jiangpeng Wei, Jianyong Zheng, Qingchuan Zhao, Xiaohua Li, Guosheng Wu.

**Formal analysis:** Jiangpeng Wei, Jianyong Zheng, Xiaohua Li.

**Funding acquisition:** Xiaohua Li.

**Investigation:** Jiangpeng Wei.

**Project administration:** Weizhong Wang, Guosheng Wu.

**Resources:** Xiaohua Li.

**Software:** Yi Yang.

**Supervision:** Dongli Chen.

**Visualization:** Guosheng Wu.

**Writing – original draft:** Jiangpeng Wei, Weizhong Wang, Qingchuan Zhao, Xiaohua Li, Guosheng Wu.

**Writing – review & editing:** Jiangpeng Wei, Qingchuan Zhao, Xiaohua Li, Guosheng Wu.

Jiangpeng Wei orcid: 0000-0001-6619-8695.
